# Potentially Inappropriate Medications Use among Older Adults with Dyslipidaemia

**DOI:** 10.3390/jcm12124063

**Published:** 2023-06-15

**Authors:** Monira Alwhaibi, Hadeel Alkofide

**Affiliations:** 1 Medication Safety Research Chair, College of Pharmacy, King Saud University, Riyadh 11149, Saudi Arabia; 2 Department of Clinical Pharmacy, College of Pharmacy, King Saud University, Riyadh 11149, Saudi Arabia; halkofide@ksu.edu.sa

**Keywords:** ageing, Beers criteria, elderly, inappropriate prescribing

## Abstract

Background: Since older patients with dyslipidemia frequently receive many prescriptions, medication errors are typical and expected in this population. This risk has increased by using potentially inappropriate medications. The 2019 Beers criteria were used in this study to investigate potentially inappropriate medication use among older individuals with dyslipidemia. Methods: A cross-sectional retrospective analysis used data from electronic medical records from an ambulatory-care environment. Patients with dyslipidemia who were older adults (>65 years old) were included. To describe and find potential determinants of potentially inappropriate medication usage, descriptive statistics and logistic regression were employed. Results: This study included 2209 older adults (age ≥ 65) with dyslipidemia. The mean age was 72.1 ± 6.0 years, and the majority of the study sample had hypertension (83.7%) and diabetes (61.7%), and around 80.0% were using polypharmacy. The prevalence of potentially inappropriate medications to be avoided among older adults with dyslipidemia was 48.6%. A high risk of potentially inappropriate medication usage was found in older patients with dyslipidemia who had polypharmacy and comorbid diabetes, ischemic heart disease, and anxiety. Conclusions: This study showed that the number of medications prescribed and the presence of concurrent chronic health conditions are important indicators of the risk of potentially inappropriate medications in ambulatory older patients with dyslipidemia.

## 1. Background

Dyslipidemia is a highly prevalent chronic condition in Saudi Arabia; around one-third of older Saudi adults have dyslipidemia [1]. Individuals with dyslipidemia are at higher risk of coronary heart disease, mortality, and poor health outcomes [2,3,4]. According to estimates, more than half of all occurrences of coronary artery disease worldwide are caused by dyslipidemia, a known risk factor for cardiovascular disease [5]. Therefore, the clinical practice guidelines urge healthcare professionals to start lipid-lowering agents [6,7]. The 2021 ESC/European Atherosclerosis Society (EAS) Guidelines for the management of dyslipidemia provide crucial patient-care guidance that aims to help healthcare providers effectively and safely lower atherosclerotic cardiovascular disease (ASCVD) risk through lipid modification, establishing and maintaining a healthy lifestyle, and early modification of their lipid-related ASCVD risk [8]. Lipid-lowering medications include statins, fibrates, bile acid sequestrants, selective cholesterol absorption inhibitors, and, more recently, PCSK9 inhibitors. As the population ages, more individuals—especially older adults—will satisfy guideline criteria for pharmacological treatment since ASCVD risk rises. For secondary prevention, statins have a proven advantage in lowering ASCVD risk across age groups. However, there is an ongoing debate over whether these medications are beneficial for primary prevention in people over 75 [9].

Dyslipidemia usually coexists with other chronic health conditions, complicating the management of the illness, and may be at high risk of polypharmacy and potentially inappropriate medications [4]. There is growing evidence that older persons with dyslipidemia frequently use multiple medications, some of which may be inappropriate. Around 60% of older persons with dyslipidemia possibly used inappropriate drugs, according to a study conducted in Saudi Arabia among 4187 older adults from ambulatory-care clinics [10]. In addition, adults with dyslipidemia have a higher rate of polypharmacy, 75% [11], putting them at risk for using potentially inappropriate medications (PIMs). PIMs use among older patients with cardiovascular diseases has negatively affected health outcomes. According to Muhlack et al. (2017) and Anfinogenova et al. (2021) [12,13], PIMs raise the risk of cardiovascular events and overall mortality. It may also result in adverse drug reactions, medication errors, hospitalization, risk of falls, and lower health-related quality of life [14,15,16,17,18,19]. A study investigated potentially inappropriate medications for people 65 and older using the adverse drug reactions reported in the Portuguese pharmacovigilance system [20]. In this study, PIMs were found in 12.8% of the 2337 reports that were considered for the analysis, and 64.7% of all adverse reaction reports were deemed serious, with hospitalization ranking as the most frequent criterion (35.1%). PIMs may also place a significant financial burden on the elderly and society. The usage of PIMs can result in a $2000 increase in cost per patient, according to a systematic evaluation of 13 published research [21].

There are no studies that have examined PIM usage in older persons with dyslipidemia, despite research among older adults showing a high prevalence of PIM use [10]. The majority of research [13,14,22,23,24,25] involved older persons with cardiovascular illnesses and did not focus specifically on older adults with a cardiovascular risk factor such as dyslipidemia. Recently published articles have emphasized the link between PIM use and cardiovascular diseases [13,14,22,23,24,25]. An analysis of 404 patients’ retrospective charts who had a history of cardiovascular illness and were admitted to the cardiology program revealed that 20% of all drugs recorded were PIMs [23]. This study identified a strong relationship between the usage of PIMs and the quantity of home medications, the gender of the patient, and the types and number of comorbidities.

There are no studies that have examined the use of PIMs in older persons with dyslipidemia. Most research focused on older persons with cardiovascular disorders in general rather than older adults with dyslipidemia [13,14,22,23,24,25]. Locally, previous research revealed alarming PIMs usage (60%) in older persons with dyslipidemia from ambulatory-care patients [10] but no study particularly looked at PIMs usage-related characteristics in this population. It is critical to know factors related to PIMs use by this population, to help identify healthcare system quality gaps and plan interventions to enhance the appropriate use of medications. Therefore, this study aimed to investigate the prevalence of PIM usage among older persons with dyslipidemia and the variables influencing PIM use. 

## 2. Methods

### 2.1. Design

A retrospective study with a cross-sectional design has been utilized. Data for older patients aged 65 years and older with dyslipidemia from the patients’ health records were used. Patients with dyslipidemia were identified using the *International Classifications of Diseases—10th edition*, Clinical Modification (ICD-9-CM) diagnostic codes. The Institutional Review Board (IRB) of King Saud University approved this study (reference number E-17-2580).

### 2.2. Data Source and Data Extraction

The data for this analysis came from the electronic health records (EHRs) of a tertiary teaching hospital in Riyadh, Saudi Arabia. It is one of the largest tertiary teaching hospitals in Saudi Arabia, with a structure that can accommodate 1200 patients and provides all general and specialty medical services.

Demographic, clinical diagnostic, and prescription-medicine data were extracted from the EHRs. In the demographics file, the patients’ dates of birth, genders, marital statuses, nationalities, and types of encounters were all listed. The clinical diagnosis file contained details regarding the clinical diagnosis obtained during inpatient and outpatient visits. ICD-9-CM codes, which are used by physicians to describe diseases according to their clinical diagnosis. Information on the prescription medications used was kept in the drug file. The encrypted patient medical-record number was used to combine the demographics, clinical-diagnosis, and prescription-medicine files into a single file. The data were treated with strict confidentiality during the whole research process.

### 2.3. Measures

PIMs use in older adults with dyslipidemia was identified according to the American Geriatric Society (AGS) 2019 updated Beers criteria [26] by applying two categories: (1) medications to avoid for most older adults and (2) medications to be used with caution. Patients were classified into two categories: (1) PIMs users (i.e., use of one or more PIMs) and (2) non-PIMs users (i.e., no PIMs use). Based on the number of PIMs prescribed, the prevalence of PIM exposure was further divided into four levels: 0 (reference, no PIM exposure), 1 (prescribed one PIM), 2 (two PIMs), and 3 or more (three PIMs or more).

Variables pertaining to patients’ sociodemographics, clinical data, and polypharmacy use were obtained from the EHRs. Sociodemographics included age in years, gender, nationality (“Saudi”, “non-Saudi”), and marital (“married”, “unmarried”) status. Chronic illnesses such as cancer, diabetes, hypertension, heart failure, chronic obstructive pulmonary disease, osteoarthritis, osteoporosis, dementia, depression, chronic kidney disease, and asthma were also independent variables. In our study, concurrent daily usage of five or more drugs was referred to be polypharmacy use. This definition of polypharmacy use has been used frequently in the literature [27]. We have calculated the average number of drugs in each patient’s medical record using this criterion.

### 2.4. Statistical Analysis

For categorical data, frequencies and percentages were used to describe the study population, whereas the mean and standard deviation (SD) were used for continuous variables. Pearson’s chi-squared tests and t-tests were used to evaluate the differences in independent variables between patients with and without PIMs. Binary logistic regression was used to examine the use of PIMs; a significance level of = 0.05 and a 95% confidence interval (CI) were used. Analyses were performed using the Statistical Analysis Software version 9.4 (SAS^®^ 9.4).

## 3. Results

### 3.1. Description of the Study Population

In this study, 2209 older adults (age ≥65 years) with dyslipidemia were identified and included. The mean age was 72.1 ± 6.0 years and the majority of the study sample had hypertension (83.7%), diabetes (61.7%), and around 80.0% were using polypharmacy. Table 1 lists the characteristics of the study sample.

### 3.2. Prevalence of PIMs and Factors Associated with PIMs Use from Bivariate Analysis

The prevalence of PIMs to be avoided among older adults with dyslipidemia was (48.6%) (Table 1 and Table 2). According to a bivariate analysis, older persons with dyslipidemia who also had coexisting hypertension, diabetes, heart failure, ischemic heart disease, anxiety, and cancer used PIMs more often than those who did not have these chronic conditions. For instance, people with anxiety used PIMs at a higher percentage than those without anxiety (67.7% vs. 48.3%, *p*-value 0.001). Additionally, older patients who used polypharmacy used PIMs more frequently than older patients who did not (55.5% vs. 21.6%, *p*-value 0.0001). 

Around 38.0% of older adults with dyslipidemia used one PIM (Table 2). The most commonly prescribed PIMs to be avoided for older adults were gastrointestinal and endocrine agents followed by antidepressants. 

### 3.3. Factors Associated with PIMs Use from Regression Analysis

The odds of PIMs use among older adults with dyslipidemia were higher in those with diabetes, ischemic heart disease, anxiety, and polypharmacy. Table 3 lists the adjusted odds ratios (AOR) and 95% confidence intervals (CI) for variables linked to PIM use. For example, those with polypharmacy use were fourfold more likely to have PIMs use compared to those without polypharmacy use (Table 3).

## 4. Discussion

Using the Beers criteria to analyze PIMs use from ambulatory care visits, this study demonstrated a high number of prescribed medications and the risk of PIM in older patients with dyslipidemia. The average number of drugs prescribed among ambulatory older persons with dyslipidemia clearly demonstrated the risk of PIMs; those who received an average of seven prescriptions had higher PIM risks than those who received an average of five prescriptions. In addition, our study found that older ambulatory patients with dyslipidemia were more likely to have PIMs when they had diabetes, ischemic heart disease, or anxiety, in addition to the higher number of prescriptions. Due to the aging population and rising life expectancy, dyslipidemia comorbidity is common. It is defined as the coexistence of two or more chronic diseases in older adults [7]. Comorbidity is linked to an increased risk of death, poor quality of life, impairments, adverse medication events, and higher PIM usage [28]. 

Our study’s PIM prevalence of 48.6% is well within the range of older persons with cardiovascular illnesses who have been reported to have PIM use ranging from 17 to 85% [13,14,22]. Anfinogenova et al., in their review article, highlighted that the most frequently administered medications to elderly patients with cardiovascular diseases are those for cardiac conditions [13]. Knowing that the prevalence of PIMs varies significantly depending on the measures and definitions used, as well as the characteristics of the studied group of patients, including clinical settings and type of comorbidities, it would be challenging to compare the findings of our study, which involved patients with dyslipidemia (i.e., a cardiovascular risk factor), to other studies for patients with cardiovascular diseases. In our analysis, cardiovascular, gastrointestinal, diuretic, and endocrine agents were the PIMs most frequently prescribed to avoid for older persons, followed by antidepressants. This finding aligns with a prospective cross-sectional study that included 428 patients receiving established treatment for cardiovascular diseases (CVDs). The vast majority of PIMs (31%) were used to treat CVDs, followed by 22.54% used to treat endocrine disorders [24].

Our findings of the high PIMs prevalence can alert prescribers to possible PIMs when managing older persons with dyslipidemia and identifying those at risk at the ambulatory visit. Prevention of polypharmacy and PIM in older adults with dyslipidemia is critical in improving the patients’ health outcomes. Some techniques supported by evidence can be used in practice to evaluate PIMs in older adults. One such instrument is the Beers criterion list. It was first published in 1991 as a list of PIMs for elderly patients [26]. The STOPP/START criterion (15) is another recommended screening method for older adults [29]. Unlike Beers, the STOPP/START criteria can be utilized as a checklist and offer a quick approach to evaluate a patient’s medication. 

PIMs and polypharmacy may hinder patients’ lipid-lowering treatment (LLT) regimen adherence. In addition, treatment adherence can be impacted by medications that need to be taken frequently [30]. Therefore, proprotein convertase subtilisin-kexin type 9 (PCSK9), an efficient LLT with a lower frequency of administration and lower possibility for polypharmacy, may increase patient adherence to LLT. The anti-PCSK9 mAbs (such as alirocumab and evolocumab), which are given every two weeks or once a month, and the twice-yearly subcutaneous dosing regimen of Inclisiran are two examples of potential LLTs [31]. Additionally, using fixed-dose coformulations rather than free combinations is another successful method for reducing polypharmacy in patients with dyslipidemia and concomitant comorbidities [32,33]. The fixed-dose combination has simplified medication taking and increased adherence to therapy. For instance, treatment with triple fixed-dose atorvastatin/perindopril/amlodipine (CTAPA) enhanced adherence more than free combinations, according to an observational retrospective study using administrative databases of three Italian health units [32]. Another randomized, controlled clinical trial compared the use of a polypill (aspirin, angiotensin-converting enzyme inhibitor, and statin) to standard care for the secondary prevention of cardiovascular death and complications after myocardial infarction and documented that the polypill had a significantly lower risk of major adverse cardiovascular events [33]. A large retrospective cohort study involving 24,020 adult subjects with hypertension from three Italian local health units, evaluated the adherence to treatment in patients who switched to the fixed-dose combination of perindopril/amlodipine versus single-pill therapy. According to this study, patients receiving perindopril/amlodipine at a fixed dose had better adherence to treatment, an increased rate of staying on therapy, and required fewer concurrent antihypertensive medications than subjects treated with single-pill therapy or a two-pill combination (*p*-value = 0.001) [34].

In the current study, older persons with diabetes, ischemic heart disease, anxiety, and polypharmacy had greater probabilities for using PIMs after adjusting for a number of patient-level factors. In other studies that have been published, comorbidities have been found to be a predictor of PIM usage [13,14,22,23,24,25,35]. The use of five or more drugs was one of the factors in this analysis that was most likely to be linked to PIM use. It is not surprising at all that taking concurrently multiple medications results in the use of PIMs. The patient is more likely to develop PIM as the number of medications increases. This result is in line with several research that found older persons who use PIMs more frequently also tended to be on polypharmacy [36,37].

### 4.1. Study Implications

In a practical outpatient context, this study offers crucial clinically relevant information to advise doctors of the risk factors for PIM in older patients with specific comorbidities. The findings discussed above can be used to understand better the factors that influence the usage of PIMs in senior populations with dyslipidemia. Healthcare practitioners’ roles may be expanded to guarantee that vital safety precautions are taken when treating older persons with dyslipidemia. Additionally, integrating pharmacy services appropriately, such as continuous medication review, can aid in avoiding inappropriate medication prescribing and use. 

### 4.2. Strengths and Limitations

We must acknowledge that this study has some limitations. The retrospective nature of the research and the fact that it was carried out in just one medical facility may have some limitations on our findings. Additionally, because it was conducted in an outpatient clinic of a single tertiary hospital in Riyadh, Saudi Arabia, generalizations about other contexts and areas are not possible from the results of this study. The rate of PIMs use may have been impacted by our patients’ use of over-the-counter medications received from outside the medical facility, and we were unable to get this information for the electronic health records. However, the comorbidity data used in this investigation was based on disease diagnostic codes; therefore, our findings are grounded in real-world data.

## 5. Conclusions

In conclusion, our study showed that the number of medications prescribed, and the presence of concurrent chronic health conditions are important indicators of the risk of PIM in ambulatory older patients with dyslipidemia. Future research to investigate the adverse health outcomes related to PIM usage and measures to justify using unneeded or high-risk drugs among this population are warranted, given the predicted rise of older people.

## Figures and Tables

**Table 1:** Characteristics of the Study Sample, Raw Percentage of Characteristics by Potentially Inappropriate Medication Use among Older Adults, Aged 65 years and above, with Dyslipidemia.

	Total		PIMs Use		No PIMs Use			
	N	%	N	%	N	%	*p*-Value	Sig.
Total	2209	100	1073	48.6	1136	51.4		
Age Mean (SD)	72.1 (6.0)		72.3 (6.0)		72.0 (6.1)		0.261	
# Rx Mean (Min, Max)	6.7 (2.9)		7.7 (8.1)		5.5 (5.8)		<0.0001	***
# Conditions (Min, Max)	3.0 (1.0)		3.1 (1.0)		2.8 (0.9)		<0.0001	***
Gender							0.022	*
Male	717	32.5	323	45.0	394	55.0		
Female	1492	67.5	750	50.3	742	49.7		
Marital Status							0.400	
Single	88	4.5	46	52.3	42	47.7		
Married	1877	95.5	895	47.7	982	52.3		
Nationality							0.757	
Saudi	2084	94.5	1012	48.6	1072	51.4		
Non-Saudi	122	5.5	61	50.0	61	50.0		
Hypertension							<0.0001	***
Yes	1849	83.7	933	50.5	916	49.5		
No	360	16.3	140	38.9	220	61.1		
Diabetes							<0.0001	***
Yes	1364	61.7	759	55.6	605	44.4		
No	845	38.3	314	37.2	531	62.8		
Heart Failure							0.044	*
Yes	18	0.8	13	72.2	5	27.8		
No	2191	99.2	1060	48.4	1131	51.6		
Ischemic Heart Disease							0.002	**
Yes	129	5.8	80	62.0	49	38.0		
No	2080	94.2	993	47.7	1087	52.3		
Asthma							0.547	
Yes	236	10.7	119	50.4	117	49.6		
No	1973	89.3	954	48.4	1019	51.6		
Osteoarthritis							0.396	
Yes	235	10.6	108	46.0	127	54.0		
No	1974	89.4	965	48.9	1009	51.1		
Osteoporosis							0.097	
Yes	239	10.8	104	43.5	135	56.5		
No	1970	89.2	969	49.2	1001	50.8		
Anxiety							<0.0001	***
Yes	106	4.8	71	67.0	35	33.0		
No	2103	95.2	1002	47.6	1101	52.4		
Depression							0.846	
Yes	32	1.4	15	46.9	17	53.1		
No	2177	98.6	1058	48.6	1119	51.4		
Cancer							0.032	*
Yes	31	1.4	21	67.7	10	32.3		
No	2178	98.6	1052	48.3	1126	51.7		
Polypharmacy							<0.0001	***
>=5	1756	79.5	975	55.5	781	44.5		
0 to 4 drugs	453	20.5	98	21.6	355	78.4		

Note: Data for 2209 older adults, age 65 years and older, with dyslipidemia. N: Number; PIMs: Potentially Inappropriate Medications; Rx: Medications; Sig: Significance; #: Number. Asterisks (*) represent significant differences in PIMs use from t-tests and Chi-square tests. *** *p* < 0.001; ** 0.001 ≤ *p* < 0.01; * 0.01 ≤ *p* < 0.05.

**Table 2:** Summary of the Findings of Potentially Inappropriate Medications to Be Avoided for Most Older Adults with dyslipidemia According to the 2019 Beers criteria.

	N	%
Average number of PIMs (SD)	0.67 (0.7)	
Average number of medications (SD)	6.70 (2.9)	
PIMs Use to be avoided		
Yes	1073	48.6
No	1136	51.4
PIMs Use to be used with caution		
Yes	884	40.02
No	1325	59.98
Number of PIMs to be avoided		
No PIM	1136	51.4
One PIM	837	37.9
Two PIM	210	9.5
Three or more PIM	26	1.2
Number of PIMs to be used with caution		
No PIM	1325	59.98
One PIM	792	35.85
Two PIM	87	3.94
Three or more PIM	5	0.23
Classification of most common PIMs prescribed		
Cardiovascular	2209	100.0
Gastrointestinal	767	34.7
Endocrine	425	19.24
Diuretic	801	36.26
Anticoagulants	28	1.27
Pain Medications (NSAIDs)	91	4.12
Antidepressants	115	5.21
Antispasmodics	5	0.23
Antipsychotics	6	1.62
Anti-infective	3	0.14
Genitourinary	2	0.09
Antiparkinsonian agents	1	0.27

Data for 2209 older adults, age 65 years and older, with dyslipidemia. N: Number; NSAID: Nonsteroidal anti-inflammatory drugs; PIMs: Potentially Inappropriate Medications.

**Table 3:** Adjusted Odds Ratios and 95% Confidence Intervals From Logistic Regression on PIM Use among Older Patients with dyslipidemia.

	PIMs Use			
	Adjusted Odds Ratio	95% CI	*p*-Value	Sig.
Total				
Age Mean	1.009	[0.993–1.025]	0.2735	
Gender				
Male	0.925	[0.752–1.139]	0.4637	
Female (Ref.)				
Marital Status				
Single	1.096	[0.701–1.716]	0.6887	
Married (Ref.)				
Nationality				
Saudi	0.939	[0.626–1.408]	0.7597	
Non-Saudi (Ref.)				
Hypertension				
Yes	0.929	[0.706–1.221]	0.5951	
Diabetes				
Yes	1.861	[1.529–2.265]	<0.0001	***
Heart Failure				
Yes	1.306	[0.419–4.073]	0.6456	
Ischemic Heart Disease				
Yes	1.531	[1.02–2.301]	0.0401	*
Asthma				
Yes	0.995	[0.729–1.358]	0.9755	
Osteoarthritis				
Yes	0.843	[0.619–1.146]	0.2753	
Osteoporosis				
Yes	0.732	[0.532–1.006]	0.0546	
Anxiety				
Yes	1.976	[1.216–3.21]	0.006	**
Depression				
Yes	0.602	[0.269–1.344]	0.2157	
Cancer				
Yes	1.756	[0.721–4.279]	0.215	
Polypharmacy				
>=5	4.027	[3.067–5.287]	<0.0001	***
0 to 4 drugs (Ref.)				

Data for 2209 older adults, age 65 years and older, with dyslipidemia. PIMs: Potentially Inappropriate Medications; Ref: Reference group; Sig: Significance. Asterisks (*) represent significant differences in PIMs use. *** *p* < 0.001; ** 0.001 ≤ *p* < 0.01; * 0.01 ≤ *p* < 0.05.

## Data Availability

The EHR dataset used during and/or analyzed during the current study is not publicly available due to our IRB policy but is available from the corresponding author upon reasonable request.
